# Identifying Patients Who May Be Candidates for a Clinical Trial of Salvage Accelerated Partial Breast Irradiation after Previous Whole Breast Irradiation

**DOI:** 10.1155/2012/937658

**Published:** 2012-12-03

**Authors:** Linna Li, Tianyu Li, Randi J. Cohen, Penny R. Anderson, Lori J. Goldstein, Richard J. Bleicher, Gary M. Freedman

**Affiliations:** ^1^Radiation Oncology, BMH Bryn Mawr, PA, USA; ^2^Biostatistics, Fox Chase Cancer Center, Philadelphia, PA, USA; ^3^Radiation Oncology, University of Maryland, Baltimore, MD, USA; ^4^Radiation Oncology, Fox Chase Cancer Center, Philadelphia, PA, USA; ^5^Medical Oncology, Fox Chase Cancer Center, Philadelphia, PA, USA; ^6^Surgical Oncology, Fox Chase Cancer Center, Philadelphia, PA, USA; ^7^Radiation Oncology, University of Pennsylvania, PCAM/TRC 4 West, 3400 Civic Center Boulevard, Philadelphia, PA 19104, USA

## Abstract

*Background and Objectives*. Accelerated partial breast irradiation (APBI) has been proposed as an alternative to salvage mastectomy for patients with ipsilateral breast tumor recurrence (IBTR) after prior breast conservation. We studied factors that are associated with a more favorable local recurrence profile that could make certain patients eligible for APBI. *Methods*. Between 1980 and 2005, 157 Stage 0–II breast cancer patients had an IBTR treated by mastectomy. Clinical and pathological features were analyzed to identify factors associated with favorable IBTR defined as unifocal DCIS or T1 ≤ 2 cm, without skin involvement, and >2 year interval from initial treatment. *Results*. Median followup was 140 months and time to recurrence was 73 months. Clinical stage distribution at recurrence was DCIS in 32 pts (20%), T1 in 90 pts (57%), T2 in 14 pts (9%), T3 in 4 pts (3%), and T4 in 9 pts (6%). IBTR was classified as favorable in 71%. Clinical stage of IBTR predicted for pathologic stage –95% of patients with clinical T1 IBTR had pathologic T1 disease at salvage mastectomy (*P* < 0.0001). *Conclusions*. Clinical stage at presentation strongly correlated with pathologic stage at mastectomy. More than 70% of recurrences were favorable and may be appropriate candidates for salvage APBI trials.

## 1. Introduction

Breast-conserving surgery and radiation therapy are the standard alternatives to mastectomy for eligible women with Stage 0, I, or II breast cancer [1, 2]. Survival outcomes are equivalent to women who undergo initial mastectomy, and the long-term rates of ipsilateral breast tumor recurrence (IBTR) are on the order of 5% to 15% [3–6]. For those who develop a clinically isolated IBTR after breast-conserving surgery and whole-breast radiation, salvage mastectomy is generally considered the standard of care [1].

Three-quarters of local recurrences are clinically solitary [7, 8], with an average size of 1-2 cm [9–14]. Therefore, many IBTR appear amenable to additional attempts at breast-conserving surgery. Breast conservation has many potential benefits on patient self-image, sexuality, and quality of life compared to mastectomy [15]. However, second attempts at breast conservation with surgery alone have been associated with high risks of second IBTR of 18–48% [13, 16–19]. Arguments against reirradiation to improve local control include concerns for radiation resistance of the recurrent tumor, acute toxicity, poor cosmesis, or risk of serious late effects. It is not possible from the few available retrospective studies of external beam boost radiation to conclude whether local control after salvage breast conservation surgery is improved by conventional re-irradiation [16, 20, 21]. However, the rationale for postexcision radiation should be the same as in the initial adjuvant setting—to address the risk for microscopic residual disease in the region of the excision cavity that exists even when surgical margins are negative. And these early reports have demonstrated low risks of complications in selected patients.

Accelerated partial breast irradiation (APBI) is radiation confined to the immediate area around an excision cavity rather than to the whole breast volume. In the setting of a clinical trial, APBI has been proposed as an alternative to salvage mastectomy for selected patients with favorable IBTR after breast-conserving surgery and whole-breast irradiation [21, 22]. APBI has the potential of improving local tumor control compared to breast-conserving surgery alone. The use of hypofractionation and the accelerated treatment time with PBI could improve the tumor control of tumors that have recurred after prior conventional 2 Gy fractionation by overcoming effects of intrinsic radioresistance, repair or rapid repopulation. APBI may reduce risk of toxicity of conventional re-irradiation by limiting the volume of breast tissue and neighboring normal tissue treated to high cumulative doses.

Proper patient selection for an IBTR of limited extent and favorable biology would be essential for treatment by APBI to have a chance of securing local control. A favorable profile of IBTR for such a trial of APBI would need to include isolated recurrences that are unifocal and limited in size so that a repeat breast-conserving surgery could obtain negative margins and maintain good cosmesis. In addition, the area of involvement would need to be limited in size without multifocality or multicentricity for a focused treatment such as APBI to have a reasonable chance for local control. Tumors with a short interval to recurrence after radiation, less than 2 years being a typical cutoff, would more likely have an aggressive biology with a poorer prognosis due to a high incidence of systemic progression [6, 19].

To better identify candidates who could have been eligible for a salvage APBI protocol, we studied the clinical and pathologic characteristics of 157 IBTRs after salvage mastectomy to identify factors associated with a favorable recurrence profile.

## 2. Materials and Methods

We retrospectively reviewed a prospective database of 3310 consecutive women with early-stage breast cancer treated with breast-conserving surgery and radiation therapy from 1980 to 2005. Patient demographics, tumor characteristics, and treatment-related information were entered prospectively and the data were maintained and updated by a single data manager. The collection, storage, and retrieval of data were done in compliance with the hospital's Institutional Review Board and the Health Insurance Privacy and Portability Act.

Inclusion criteria for this study were primary breast cancer; American Joint Committee on Cancer 6th edition initial cancer stages 0, I, or II [23]; initial treatment with whole breast radiation therapy; a clinically isolated IBTR; and treatment for IBTR by mastectomy. Exclusion criteria included male breast cancer, T3-T4 disease, Stage IV disease, mastectomy for initial treatment, and no radiation therapy as part of the patient's initial treatment. The study population consisted of 157 patients who met the above criteria. All patients were treated initially by breast-conserving surgery followed by whole-breast radiation (46–50 Gy), with or without regional nodal radiation, and a boost to the tumor bed (10–18 Gy). The total dose was generally determined by the final margin status after lumpectomy: 60 Gy for a negative margin, 64 Gy for a close margin, and 66 Gy for a positive final margin.

 The study endpoint was a classification as a favorable IBTR defined as an isolated first site of recurrence; unifocal; invasive or in situ; less than or equal to 2 cm in size; no skin involvement; and more than 2 years from initial treatment. Chi-square test, Wilcoxon's test, and generalized estimating equations were used for univariate and multivariate analyses.

## 3. Results

The characteristics of the 157 patients in the study population are summarized in Table 1. At the time of their initial diagnosis and treatment, 75% of patients were between the ages of 40 years and 70 years. Median age was 49 years. Patients were approximately equally divided between pre- and postmenopausal status and laterality of the breast cancer. Median followup from initial treatment was 140 months.

The characteristics of the 157 IBTR are shown in Table 2. Median time to IBTR was 73 months. The interval to recurrence was ≤2 years in 8% and >2 years in 92%. IBTR was confined to the same quadrant as the initial tumor in 55% of patients, a different quadrant in 33%, was diffuse or multicentric in 6%, and skin was involved in 3%. The T stage of the IBTR was T1 in 57%. Histologically, most patients presented with invasive ductal carcinoma (77% of primary tumors and 57% of IBTR). Receptor status was considered hormone sensitive (ER or PR positive) or insensitive (ER and PR negative). This was available for only 39% of the IBTR compared to 72% of the initial tumors. Patients uniformly did not undergo re-dissection of the axilla so that pathologic N stage is unknown at time of recurrence.

The differences in characteristics between the initial tumor compared to the subsequent IBTR are shown in Table 3. There were more palpable tumors at time of initial diagnosis (55%) compared with IBTR (41%). The method of detection was physical examination only in approximately 20% at time of initial diagnosis and IBTR. More IBTR were detectable on mammogram alone compared to the initial tumors (55% versus 40%). Pathologic T stage at initial diagnosis and clinical T stage of IBTR were predominantly T1, 65% and 57%, respectively. Among patients with known receptors, the percentage of hormone sensitive tumors was 78% of initial tumors and 67% of IBTR (Table 3). For patients initially hormone sensitive, the IBTRs was hormone sensitive in 37 of 41 (90%). For patients known to be initially hormone insensitive, the IBTR was sensitive in 9 of 20 (45%).

Among the 90 patients with a clinical T1 IBTR, pathology from salvage mastectomy was available for 75 patients. In 71 of 75 patients (95%) with clinical T1 IBTR the pathologic tumor size was also <2 cm. For those with pathologic data, the IBTR were clinical stage T1 or DCIS in 77% and pathologic Tis or T1 in 70%. The median tumor size at time of both initial diagnosis and IBTR was 1 cm. The characteristics of the initial tumor versus the IBTR were analyzed to determine predictors of pathologic T1 size at time of salvage mastectomy. The results of the multivariate analysis are shown in Table 4. There was no significant correlation between the studied initial tumor characteristics and subsequent pathological size of IBTR. Clinical T stage at recurrence was the only independent predictor of having a T1 pathologic recurrence stage.

## 4. Discussion

Approximately 10–20% of patients with Stage I or II invasive breast cancer will develop an IBTR by 10 years after breast-conserving surgery and RT [3–6, 24–28]. In general, IBTR rates have been decreasing due to improvements in patient selection for initial treatment with breast-conserving surgery and whole breast radiotherapy, surgical and radiation techniques, and the use of systemic therapy [29, 30]. Our study population of only 157 IBTR from an initial population of over 3,000 patients (less than 5%) after a median followup of 140 months is consistent with this reported decreasing risk of IBTR in other studies.

Current recommendations for surveillance of patients following breast-conserving therapy include monthly patient self-examination, examination by a physician every 4 to 6 months for 5 years and then annually, and mammography 6 months after radiation and then annually [1]. In the current study, we found that 80% of IBTR were detectable on mammography, and the median size was 1 cm. Approximately 80% of IBTR were Tis or T1. This relatively early stage of detection of IBTR supports the current recommendations for surveillance. We have insufficient numbers of patients with IBTR T2 or larger to analyze for significant characteristics that could be prospectively identified. If there were a common independent factor that predicted for a large size of IBTR with current methods of physical examination and mammography, then a more intensive surveillance could be recommended for such patients.

Mastectomy is the standard treatment for patients with a clinically isolated IBTR after whole-breast irradiation [1]. Salvage mastectomy is associated with local control rates of approximately 85–95% [8, 13, 17, 19, 31–34]. A change in this paradigm for salvage therapy needs to be approached with caution so that new treatments for IBTR with lower rates of local control do not become commonplace. However, in the setting of a clinical trial, for women who find mastectomy unacceptable or are medically poor candidates, identification of other salvage treatment modalities for IBTR may be appropriate. However, second attempts at breast conservation with surgery alone have been associated with high risks of second IBTR of 18–48% [13, 16–19].

There is a limited published experience with giving further RT after prior whole-breast irradiation after salvage breast-conserving surgery for IBTR. In the study of Kurtz et al., 11 of 50 patients who had recurrences away from the original tumor bed were given additional radiation after wide excision [16]. Second local failures occurred in 36% of the patients treated with further irradiation, compared with 31% of those treated with wide local excision alone. Deutsch and colleagues reported on a series of 39 women treated for IBTR by repeat wide local excision and treatment to an electron field around the lumpectomy bed with an additional 50 Gy [20]. The subsequent second IBTR rate was 23%. There were no reported serious sequelae from the additional radiation.

In the setting of a clinical trial, APBI has been proposed as an alternative to salvage mastectomy for selected patients with favorable IBTR after breast-conserving surgery and whole-breast irradiation [21, 22]. Hannoun-Levi et al. reported on 69 patients with IBTR who were treated by a second breast-conservation surgery and interstitial brachytherapy [21]. The incidence of second IBTR was 23% at 5 years. Factors associated with better local control were an interval to recurrence of 36 months or greater and use of a greater number of catheters for the implant. This suggests that improved methods of radiation technique that optimize dose coverage may lead to better rates of local control in future studies.

In addition to optimized APBI techniques, improved patient selection could result in improved rates of local control after salvage breast conservation for IBTR. A favorable profile of IBTR for APBI would need to be isolated, unifocal, and limited in size. Selection of tumors with a longer interval to recurrence would also include IBTR with less aggressive biology and patients with better chances for long-term survival. We identified approximately 70% of patients with these favorable IBTR characteristics after initial breast-conserving surgery and whole-breast radiation. The clinical estimation of tumor size was the most significant independent factor predictive of having pathologically confirmed favorable IBTR at salvage mastectomy. This favorable subset of patients could be a pool of eligible candidates for a clinical trial of salvage breast conservation in this setting.

The Radiation Therapy Oncology Group is currently studying salvage breast-conserving surgery and APBI in this favorable subset of patients. 3D conformal external beam radiation will treat the second surgical cavity plus margin. Patient selection includes IBTR 3 cm or less in size, without imaging evidence of multicentricity, and an interval to recurrence of greater than 1 year. Our data suggests that over 70% of patients with IBTR will be eligible for enrollment given that our selection criteria for most favorable IBTR are more strict than the RTOG trial eligibility.

## Figures and Tables

**Table 1:** Characteristics of 157 patients at time of initial diagnosis.

Age (yrs)	
Median	49 (25–77)
<40	24 (15%)
40–54	75 (48%)
55–69	43 (27%)
70+	15 (10%)
Menopause status	
Pre	73 (47%)
Peri	9 (6%)
Post	75 (48%)
Race	
White	142 (90%)
Black	12 (8%)
Breast laterality	
Right breast	75 (48%)
Left breast	82 (52%)
Followup	
Median	140 mos
Range	12–296 mos

**Table 2:** Characteristics of 157 local recurrences after whole-breast irradiation.

Clinical T stage	
Tis	32 (20.4%)
T1	90 (57%)
T2	14 (9%)
T3/4	13 (9%)
Unknown	8 (5%)
Pathologic T stage	
Tis/T1	110 (70%)
T2/3/4	14 (9%)
Unknown	33 (21%)
Location	
Unifocal	139 (89%)
Multifocal/diffuse	9 (6%)
Skin involvement	5 (3%)
Unknown	4 (2%)
Time to recurrence	
Median (range)	73 mos (7–265)
≤24 months	12 (8%)
>24 months	138 (92%)

**Table 3:** Comparison of tumor characteristics between initial and recurrent tumors.

	Initial	Recurrence
Detection method		
Physical examination	32 (20%)	35 (22%)
Mammogram	63 (40%)	87 (55%)
PE + Mammogram	62 (35%)	29 (19%)
T stage	(pathologic)	(clinical)
Tis	20 (13%)	—
1	103 (65%)	90 (57%)
2	34 (22%)	14 (9%)
3/4	—	13 (9%)
N stage		
0	136 (87%)	—
1	21 (8%)	—
2	8 (5%)	—
Tumor size		
Median	1.0 cm	1.0 cm
Range	0.2–4.5 cm	0.2–5.5 cm
Histology		
DCIS	20 (13%)	34 (22%)
Invasive ductal	121 (77%)	89 (57%)
Invasive lobular	9 (6%)	10 (6%)
Grade		
1	9 (6%)	—
2	31 (20%)	—
3	51 (33%)	—
EIC		
Positive	18 (12%)	—
Negative	73 (47%)	—
Receptor status		
ER or PR +	88 (56%)	41 (26%)
ER and PR −	25 (16%)	20 (13%)
Margins		
Negative	106 (67.5%)	—
Close	14 (9%)	—
Positive	6 (3.8%)	—

**Table 4:** Predictors of pathologic T1 size of recurrence from multivariate analysis.

Characteristics	*P* value
Age	0.85
Initial T stage	0.24
Initial grade	0.17
Initial histology	0.64
Initial necrosis	0.10
Initial EIC	0.89
Initial margin status	0.57
Initial receptor status	0.92
Recurrence method of detection	0.88
Recurrence clinical stage	<0.001
